# Clinical and Paraclinical Predictors of Survival in Amyotrophic Lateral Sclerosis: Results from a Three-Year Longitudinal Cohort Study

**DOI:** 10.3390/medsci13030170

**Published:** 2025-09-03

**Authors:** Anca Motataianu, Laura Barcutean, Ioana Ormenisan, Medeea Roman, Rodica Balasa, Zoltan Bajko, Mihai Dumitreasa

**Affiliations:** 1 Neurology Department, University of Medicine, Pharmacy, Science and Technology “George Emil Palade” Targu Mures, Gh. Marinescu Str., No. 38, Mures, 540142 Targu Mures, Romania; motataianuanca@gmail.com (A.M.); rodica.balasa@umfst.ro (R.B.); zoltan.bajko@umfst.ro (Z.B.); 2 Neurology 1 Clinic, Emergency Clinical County Hospital Targu Mures, 540136 Targu Mures, Romania; ioana.ormenisan@gmail.com (I.O.); r.medeea@yahoo.com (M.R.); mihai.du96@gmail.com (M.D.)

**Keywords:** Amyotrophic Lateral Sclerosis, survival analysis, ALSFRS-R, disease progression rate, clinical phenotypes, progression patterns, respiratory parameters

## Abstract

Background: Amyotrophic Lateral Sclerosis (ALS) is a heterogeneous neurodegenerative disorder with highly variable progression and survival. Identifying early prognostic indicators is essential for patient stratification and management. Objectives: To evaluate clinical, respiratory, and functional predictors of survival in a prospective cohort of ALS patients over a three-year period. Methods: A cohort of 44 ALS patients was followed from 2022 to 2025. Demographic and anthropometric characteristics, clinical data including ALS subtypes and phenotypes, site of onset, revised ALS functional rating scale (ALSFRS-R) and subscores, ALSFRS-R progression rate, time to diffusion and generalization, spirometric parameters, and progression patterns were assessed. Survival analysis was performed using Kaplan–Meier estimates and univariate and multivariate Cox proportional hazard regression analysis. Results: The overall median survival time was 53 months. Univariate Cox regression revealed that older age at onset, shorter diagnostic delay, lower respiratory function, lower vitamin D levels, and rapid vertical progression were associated with reduced survival. Bulbar-onset phenotype and rapid disease progression rate (ΔPR) were significant predictors of mortality. Specific ALSFRS-R subscores also showed prognostic relevance. A longer time to diffusion as well as a longer time to generalization were significantly associated with prolonged survival. Multivariate analysis confirmed the independent prognostic value of ΔPR, time to diagnosis, and ALSFRS-R swallowing and handwriting subscores. Conclusions: This study supports the prognostic value of previously studied clinical and paraclinical markers in ALS and proposes novel predictors, ALSFRS-R handwriting, and time to diffusion, which require further validation in larger prospective cohorts.

## 1. Introduction

Amyotrophic Lateral Sclerosis (ALS) is a progressive, neurodegenerative disease with poor survival outcomes, affecting the motor neurons from the brain cortex and spinal cord, leading to impairment of voluntary movements, swallowing, speaking, and finally, respiratory failure [1].

Understanding the progression of ALS presents several challenges, including the variability in survival rates, the heterogeneity of symptoms, and the existence of distinct clinical subtypes. With life expectancy typically ranging from 2 to 5 years and frequent diagnostic delays, it is crucial not only to recognize the clinical signs and symptoms promptly and classify them according to established diagnostic criteria, but also to identify both clinical and paraclinical prognostic markers that may improve and extend the patients’ quality of life. Despite this, some patients exhibit long-term survival, living 10 to 20 years or longer after diagnosis [2,3]. The incidence and prevalence of ALS vary globally depending on geographic region, age, sex, and race. The individuals most at risk of developing the disease are males, Caucasians, those over the age of 60, and those with a family history of ALS [4]. A higher prevalence rate has been recorded in Europe and North America, ranging from 1.71 to 1.89 per 100,000 inhabitants, while in Europe, the highest incidence rates have been observed in Scotland, Austria, and Denmark (5.55 per 100,000 inhabitants per year) [4].

To enhance the understanding of ALS progression and improve patient stratification based on survival outcomes, various studies have identified demographic, clinical, and paraclinical prognostic factors, which have been incorporated into prognostic models [5,6,7]. However, the marked heterogeneity of ALS in terms of clinical presentations, progression patterns, and underlying genetic and molecular mechanisms pose a significant challenge to the development of universally applicable models [8,9,10]. Moreover, while some clinical and paraclinical prognostic indicators, such as age at onset, site of onset, respiratory parameters, and neurofilament light chain, are well established [11,12,13,14,15], gaps remain in identifying reliable, standardized biomarkers for survival prediction. A more comprehensive understanding of these prognostic variables is essential to personalize disease management, guide therapeutic decisions, and optimize clinical trial design [16].

We performed an observational, longitudinal, single-center study on ALS patients with the following aim: to identify possible clinical and paraclinical factors associated with survival over a three-year period of regular follow-ups.

## 2. Materials and Methods

### 2.1. Study Design and Participants

The study evaluated the survival of forty-four patients diagnosed with sporadic ALS in the Neurology Department of Târgu Mures Emergency Clinical County Hospital (a tertiary referral center). All the patients were diagnosed with ALS in the last 12 months (2021–2022). The patients were enrolled in the study between August 2022 and November 2022, when the clinical, neurophysiological, and paraclinical evaluations were performed. Death or tracheotomy was employed as the outcome measure to assess mortality across various time points (12–24–36 months) and the last recorded death/tracheostomy recorded was 31 March 2025. For patients who were unable to return to the center for follow-up, information regarding death or tracheotomy was obtained through telephone interviews.

Inclusion criteria stipulated the following: (1) fulfillment of the El Escorial-Arlie House criteria for definite or probable ALS diagnosis, with exclusion of possible ALS cases, as well as compliance with Awaji criteria [17,18]; (2) age ≥ 25 years old; (3) absence of familial ALS history. Exclusion criteria comprised the following: (1) diagnosis of other neurodegenerative diseases; (2) subjects whose Mini-Mental State Examination (MMSE) scores were <24; (3) prior history of symptomatic pulmonary disease unrelated to ALS; (4) presence of neurological conditions or comorbidities potentially interfering with ALS progression assessment. Additionally, all ALS patients received ALS standard therapy approved in Europe, which is 100 mg/day of riluzole.

### 2.2. Data Collection and Procedures

The following variables were documented during study clinical assessment: (1) baseline demographic and anthropometric data including gender, height, body mass index (BMI), and alcohol and smoking habits; (2) age at ALS onset (years); (3) age at ALS diagnosis (years); (4) time from onset to diagnosis, defined as the time from the onset of the first symptom to ALS diagnosis (months); (5) time from onset to treatment (months); (6) clinical subtype of ALS onset (spinal or bulbar); (7) the site of ALS onset (cervical vs. lumbar vs. bulbar); (8) ALS clinical phenotypes; (9) baseline score on the revised ALS Functional Rating Scale (ALSFRS-R); (10) the ALSFRS-R progression rate (ΔPR) was calculated as the change in ALSFRS-R score from diagnosis to the date of study visit, using the following formula: 48—[(ALSFRS-R at diagnosis—ALSFRS-R at study visit)/duration of symptoms (in months)] [19]; (11) time to diffusion, defined as the time from the onset of the first symptom to extension to next region; (12) time to generalization, defined as the time to spread of clinical signs from an initial spinal (cervical, thoracic, or lumbosacral) or bulbar onset to involvement of both regions, considering all lower and upper motor neuron signs as defined by the El Escorial criteria across the four body regions and the spreading of the clinical signs from spinal or bulbar localization to both [20]; (13) pattern of progression (horizontal vs. vertical); (14) survival time, defined as time (months) from symptom onset to the date of death or tracheostomy to the censoring date (31 March 2025). Tracheostomy is a marker of end-stage respiratory failure in ALS and was therefore considered as a clinically significant endpoint.

Patients were divided according to BMI into underweight (<18.5 kg/m^2^), normal weight (18.5–24.9 kg/m^2^), overweight (25.0–29.9 kg/m^2^), and obese (≥30 kg/m^2^) [21].

Functional neurological impairment was evaluated using the revised ALS Functional Rating Scale revised (ALSFRS-R) and manual muscle testing (MMT) [22]. The ALSFRS-R assessment included four subscores calculated for bulbar function (ALSFRS-R-B), lower limb function (ALSFRS-R-LL), upper limb function (ALSFRS-R-UL), and respiratory function (ALSFRS-R-R), with a maximum total score of 48 points [22]. Baseline ALSFRS-R scores were recorded at the time of diagnosis.

At the diagnosis, patients were classified into three ALS phenotypes based on clinical and electromyographic findings: (1) classical or typical ALS, characterized by predominant lower motor neuron (LMN) signs in two or more body regions, along with upper motor neuron (UMN) involvement in at least one region; (2) LMN-predominant ALS, including the flail arm and flail leg variants; (3) bulbar-onset ALS, defined by initial and progressive bulbar symptoms such as dysarthria or dysphagia [23]. The primary lateral sclerosis phenotype was not included in our classification as there were no patients who met the diagnostic criteria.

The progression of ALS was assessed by evaluating the spread of muscle atrophy or weakness beyond the initial site of onset, using clinical, electrophysiological, and anamnestic data. ALS patients were classified into two groups based on the direction of motor neuron degeneration from the onset site: (1) horizontal spreading pattern (HSP), characterized by progression from the cervical region to the contralateral cervical region or from the lumbar region to the contralateral lumbar region; (2) vertical spreading pattern (VSP), where degeneration spreads from the cervical or lumbar region to the ipsilateral upper or lower limb, from the bulbar region to the lumbar/cervical region, or from the cervical/lumbar region to the bulbar region [24].

Each patient underwent psychological evaluation using the Frontal Assessment Battery (FAB) and Beck Depression Inventory (BDI). Executive functions were assessed using the FAB, which includes six subtests designed to evaluate distinct processes controlled by the frontal lobes. These subtests address conceptualization, mental flexibility, motor programming and executive control of actions, resistance to interference, self-regulation, inhibitory control, and environmental autonomy. Each FAB subtest is scored on a scale from 0 to 3, with a score of 0 indicating either an absence of response or an inappropriate answer. The individual subtest scores are summed to yield a total FAB score, ranging from 0 to 18 [25,26]. The BDI was used to assess depressive symptoms, with scores greater than 10 indicating the presence of depression [27].

Pulmonary function test was evaluated by spirometry in all patients, conducted with patients in the upright-seated position. The parameters measured included forced vital capacity (FVC), forced expiratory volume in one second (FEV_1_), peak expiratory flow (PEF), maximal mid-expiratory flow rate (MMEFR), forced expiratory flow at 25–75% (FEF25–75%), vital capacity (VC), and FEV_1_/FVC ratio, using a standardized spirometer. Predicted values for each parameter were calculated based on the reference equations established by Goldman and Becklake. These predicted values were subsequently used to determine the percent predicted values for upright FVC, FEV_1_, VC, FEF25–75%, and MMEFR [28,29].

Blood samples for serum 25-hydroxyvitamin D (25(OH)D) determination were collected in the morning, after an overnight fast, under standardized conditions using serum-separating tubes. Samples were processed within 2 h of collection and analyzed by chemiluminescence immunoassay. Creatine kinase and blood creatinine levels were also measured as part of the routine blood testing, but were not included in the survival analysis due to the relatively small cohort size and the focus on clinical prognostic factors.

All patients signed the informed consent before the initial study visit, and institutional review boards approved this study (approval no 13472/21 June 2022, Ethical Committee of the Târgu Mures Emergency Clinical County Hospital). The research strictly adhered to pertinent guidelines and regulations, aligning with the principles of the Declaration of Helsinki.

### 2.3. Statistical Analysis

The continuous variables were tested for normality using the Shapiro–Wilk test. The variables with parametric distribution were reported as the mean (standard deviation, SD) and the non-parametric variables were reported as the median (interquartile range, IQR). Data comparison was performed according to normality distribution. Parametric data were assessed using Student’s *t* test, after assessing for the quality of variances using Levene’s test. When variances were unequal (*p* < 0.05), Welch’s correction was applied. For non-parametric variables we applied Mann–Whitney U test. Categorical variables were compared using the Chi-square test with Fisher’s exact test when expected frequences were equal or below 5.

Data from ALS-FRS-R (each item having ranges from 0 to 4) was coded based on the number of patients that experienced the events: under 5 patients the variable was added to another subgroup.

The survival time was defined as the time interval in months from symptom onset to either moment of death/tracheostomy or end of study (last follow up, database lock 31 March 2025), with events coded using a binary censoring variable (1). The Kaplan–Meier survival cures were plotted and compared using the log-rank test. Data was expressed as mean/median with 95% CI; several subgroups had censored data or small sample sizes which limited the ability to calculate the upper bound of CI. These values were amended as NA (not available). The Cox proportional hazard regression analysis (univariate and multivariate) was conducted in order to identify predictors of survival in ALS patients based on demographic and clinical variables. Data was presented as HR and 95% CI. The variables with a *p* value < 0.07 in univariate analysis were considered for inclusion in multivariate Cox regression models when supported by clinical relevance. We performed univariate Cox analysis for each predictor. The variables that returned statistical significance were considered to be added in the multivariate models. Due to the small sample size and low events in some of the subgroups, we further assessed the data using Schoenfeld residuals, followed by Firth’s penalized likelihood Cox regression in order to reduce the estimation bias. Before creating the models, we assessed for the multicollinearity among the predictors by using variance inflation factor analysis (VIF). The predictors that had a value close to 5 were excluded from their respective models in order to ensure stability. We devised four models based on the types of the predictors: (1) age at onset, onset to diagnosis; (2) paraclinical: FVC, FEF25–25, and vitamin D; (3) progression pattern, clinical phenotype, ΔPR, diffusion time, and generalization time; (4) ALS-FRS-R-salivation, ALS-FRS-R-swallowing, ALS-FRS-R-handwriting, and ALS-FRS-R-orthopnea.

All statistical analysis was performed in R version 4.2.2. (R Foundation for Statistical Computing, Vienna, Austria), using the following packages: survival (v. 3.5.5.), survminer (v. 0.5.0), ggplot2 (v. 3.5.2), ggsurvplot, dplyr (v. 1.1.2), coxphf (v. 1.13.4), broom (v. 1.0.8), and RcmdrPlugin (2.8.0) Statistical significance was set at *p* < 0.05.

## 3. Results

### 3.1. Clinical, Paraclinical and Demographic Characteristics of the Cohort

The demographic, clinical, and paraclinical characteristics of the cohort (*n* = 44) are presented in Table 1. There were no significant differences between gender in terms of age at diagnosis (58.75 vs. 57.53 years, *p* = 0.76), or age at symptom onset (57.06 vs. 56.39 years, *p* = 0.86). A slight trend was noted in diagnostic delay (14.5 vs. 9 months, *p* = 0.26) and treatment (18.5 vs. 9.5 months, *p* = 0.14) in female patients compared to male but without reaching statistical significance. The number of deaths during follow-up were similar between genders (11 vs. 17, *p* = 0.74). ALS-FRS scores were similar across groups (39.5 vs. 39, *p* = 0.64).

Respiratory parameters (FVC, FEV_1_, FEV_1_/FVC, PEF, and FEF25–75) showed no statistically significant differences between groups. The BDI and FAB scores showed no significant sex-based differences. Smoking and alcohol consumption were low across the cohort. Functional times (time to diffusion and time to generalization) did not significantly differ across genders.

Regarding the differences between the environments, rural vs. urban, no statistically significant differences were noted in the analyzed cohort. Smoking and alcohol consumption were low across both cohorts, limiting power to detect differences.

### 3.2. Kaplan–Meier Survival Analysis and Survival Curves

**Figure 1:**
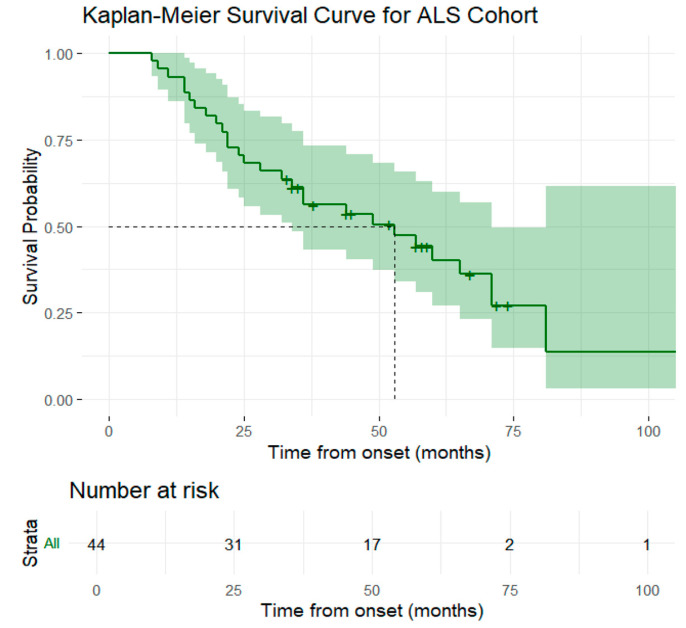
Graphical representation of the Kaplan–Meier survival curve for the entire ALS cohort. The solid green line represents the estimated survival probability, the shaded green areas indicate the 96% CI, the dashed line shows the median survival time. The table below the curve reports the number of patients at risk.

Out of the 44 patients included in the study, 28 (63.64%) met the endpoint during the study follow-up. We performed Kaplan–Meier analysis using the confirmed time of death as the event, calculated in months, from the onset of the symptoms. The summary is presented in Table 2. The survival curves did not reach 50% and the median survival time could not be estimated from the reported data; therefore, the restricted mean and 95% CI was reported for observational purposes. The overall median survival time for the entire cohort was 53 months (Figure 1). We found no statistically significant differences in survival across gender, environment, BMI, and age at the onset of the symptoms. The patients from urban environment had longer median survival times compared to those from rural environments (65 months vs. 34 months), but without reaching statistical significance (*p* = 0.2).

**Figure 2:**
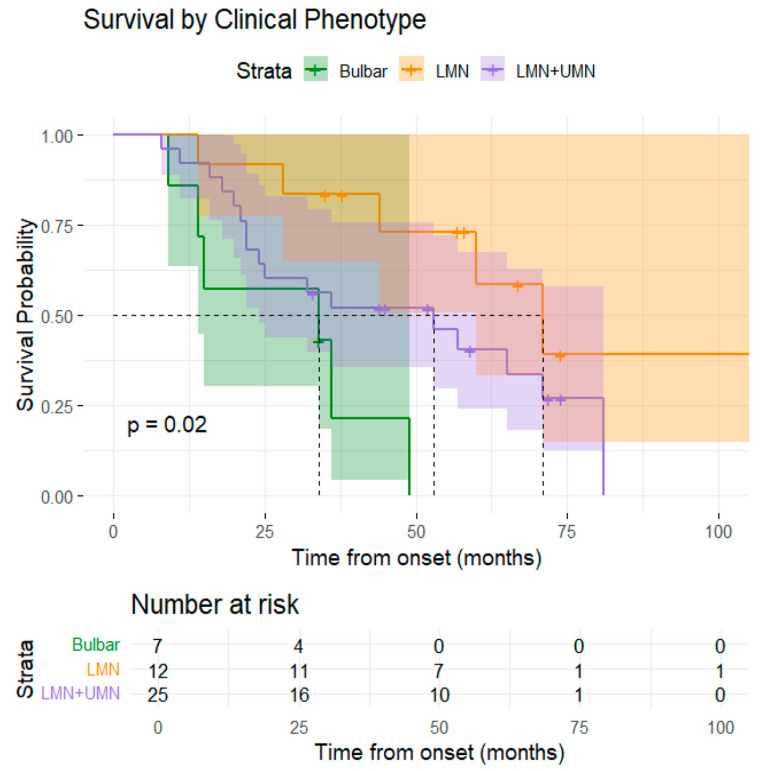
Graphical representation of the Kaplan–Meier survival curve by clinical phenotype.

**Figure 3:**
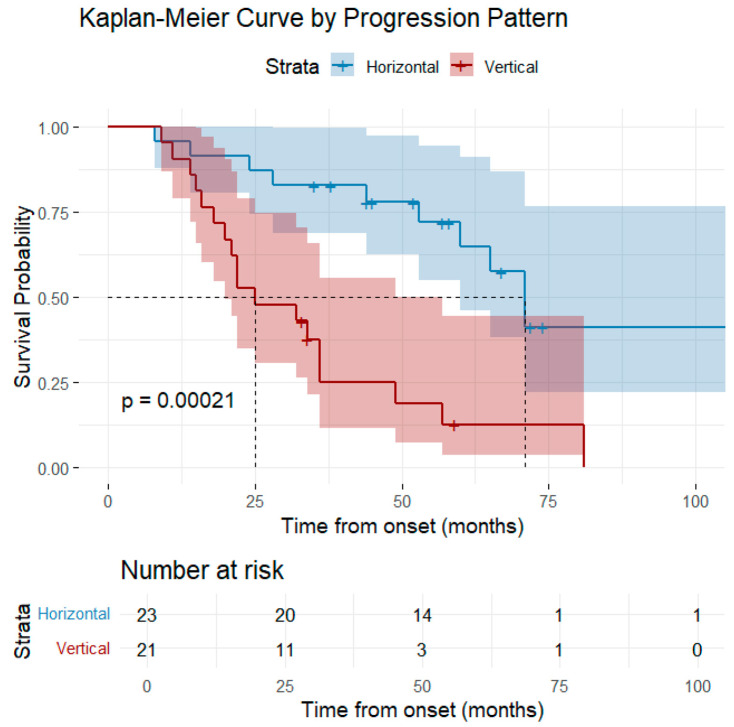
Graphical representation of the Kaplan–Meier survival curve by progression pattern.

The survival analysis based on clinical phenotype was statistically significant (*p* = 0.02). Patients with LMN-predominant ALS phenotype at onset had the longest median survival time (71 months), followed by classical phenotype (53 months) and by bulbar-onset phenotype (34 months) (Figure 2). The progression pattern was strongly associated with survival rates (*p* < 0.0002). Patients with a horizontal pattern of progression had a significantly longer median survival time compared to the patients with a vertical pattern of progression (71 months vs. 25 months) (Figure 3).

**Figure 4:**
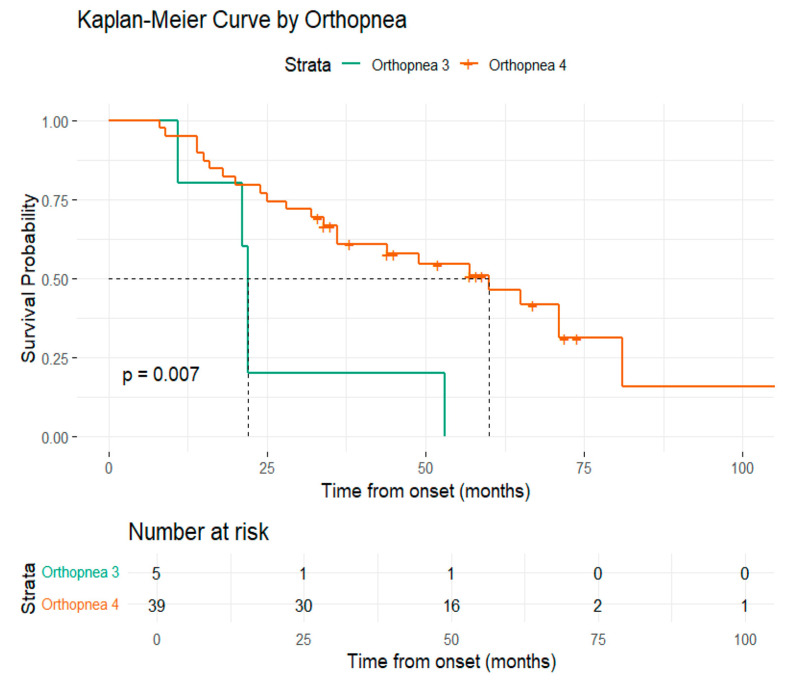
Graphical representation of the Kaplan–Meier survival curve by orthopnea.

**Figure 5:**
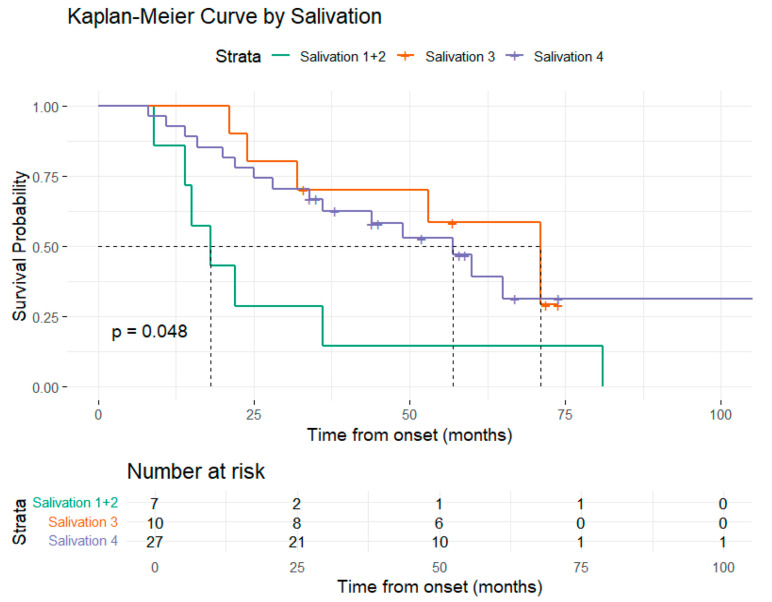
Graphical representation of the Kaplan–Meier survival curve by salivation.

**Figure 6:**
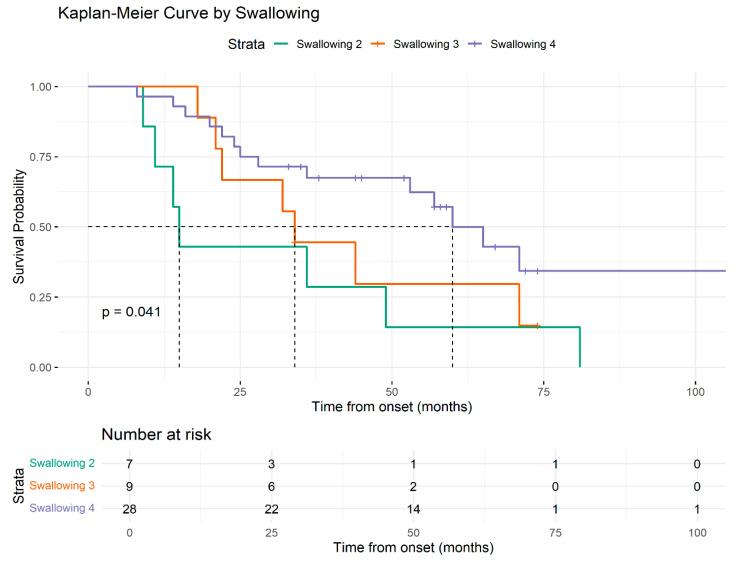
Graphical representation of the Kaplan–Meier survival curve by swallowing.

Additional survival analysis based on the ALSFRS-R functional scores demonstrated associations for the following subdomains: orthopnea, salivation, and swallowing. The presence of ALSFRS-R-orthopnea score of 3, compared to 4, demonstrated a statistically significant shorter survival rate (22 months vs. 60 months). Similarly, for ALSFRS-R-salivation and swallowing, median survival increased progressively with higher scores (Figure 4, Figure 5 and Figure 6).

### 3.3. Univariate Cox Regression Analysis

In the univariate Cox regression analysis (the summary is presented in Table 3), the older age at onset had a higher hazard of mortality, but the result was borderline significant (HR: 1.03; 95% CI 0.99–1.07, *p* = 0.06). Additionally, a shorter time from the symptom onset to diagnosis was associated with a higher hazard of mortality (HR: 0.97, 95% CI 0.94–0.99, *p* = 0.02).

A higher FVC (HR: 0.98; 95% CI: 0.97–0.99, *p* = 0.004), FEV_1_ (HR: 0.98; 95% CI: 0.973–0.994; *p* = 0.003), PEF (HR: 0.98; 95% CI: 0.969–0.996; *p* = 0.013), and FEF2575 (HR: 0.98; 95% CI: 0.976–0.996; *p* = 0.011) revealed a statistically significant association with overall survival in our cohort. The FEV/FVC ratio was not associated with survival.

Higher vitamin D levels were associated with an improved survival in our cohort (HR = 0.92, 95% CI: 0.87–0.99, *p* = 0.023).

ΔPR was significantly associated with an increased risk for death in the selected cohort (HR 3.44, 95% CI 2.15–5.51, *p* < 0.001).

Vertical disease progression was statistically significantly associated with a shorter survival, compared to horizontal progression (HR = 4.23, 95% CI: 1.87–9.57, *p* < 0.001).

The clinical phenotype was statistically significant associated with survival (*p* = 0.002). Compared to the patients that presented at the onset an LMN-predominant phenotype, the patients with bulbar-onset had a shorter survival (HR = 5.30, 95% CI: 1.53–18.33, *p* = 0.008) while the patients with a classic phenotype had a non-statistically significantly trend toward increased risk (HR = 2.18, 95% CI: 0.80–5.94, *p* = 0.12).

A longer time to diffusion, as well as a longer time to generalization, were significantly associated with prolonged survival (HR = 0.84, 95%CI: 0.79–0.91, *p* < 0.001; HR = 0.91, 95% CI: 0.87–0.95, *p* < 0.001).

Regarding the ALS-FRS subscores, the following results were statistically significant: patients with higher ALS-FRS-salivation scores (3 and 4) had a reduced hazard of death (HR = 0.30, 95% CI: 0.10–0.94, *p* = 0.03 and HR = 0.36, 95% CI: 0.14–0.91, *p* = 0.03), compared to those with scores 0–2; patients with higher ALS-FRS-swallowing score (4) had a reduced hazard of death (HR = 0.32, 95% CI: 0.12–0.82, *p* = 0.01) compared to those with score of 2; patients with higher ALS-FRS-handwriting score (4) had a reduced hazard of death (HR = 0.37, 95% CI: 0.14–0.98, *p* = 0.04) compared to those with a score of 2.

### 3.4. Multivariate Cox Regression Analysis

For all the variables included in the univariate Cox regression (after we adjusted for multicollinearity by VIF and removed variables over 5) we used Schoenfeld residuals to assess hazards. The global Schoenfeld test was statistically significant (*p* < 0.001), indicating that the model does not follow the proportional hazard assumption. Therefore, we used multivariate Cox regression with Firth’s correction. The Schoenfeld values are summarized in Table 3.

The first model evaluated the impact of age at onset and the time from onset to diagnosis (months) upon patient survival (likelihood ratio test, *p* = 0.01; Wald test, *p* = 0.02). An older age at onset was associated with an increased hazard for death (HR = 1.03, 95% CI: 0.99–1.07, *p* = 0.057), but this was not statistically significant. A delay in diagnosis (months) was associated with a better survival (HR = 0.97, CI: 0.94–0.99, *p* = 0.02) in our studied cohort.

The second model evaluated the impact of paraclinical parameters on patient survival in ALS patients (likelihood ratio test, *p* = 0.03; Wald test, *p* = 0.04). As a group, the predictors account for model variability, but without independent prediction power (all *p* > 0.05). In the third model, ΔPR remained a significant predictor of mortality (HR: 2.86, 95% CI: 1.86–4.68, *p* < 0.001). The fourth model, which evaluated the ALS-FRS-R subdomains, included salivation, swallowing, handwriting, and orthopnea, was statistically significant (likelihood ratio test, *p* = 0.002; Wald test, *p* = 0.01). The ALS-FRS-R swallowing and handwriting scores of 4 were associated with a reduced mortality risk (HR = 0.16, 95% CI 0.04; 0.71; HR = 0.19, 95% CI: 0.04–0.78, *p* = 0.02). The other subscores were not statistically significant for the presented cohort. The data is summarized in Table 4.

## 4. Discussion

In our cohort, no significant differences were observed between genders in terms of demographic, clinical, or paraclinical characteristics. A recent study investigating ALS prognosis reported that male patients tend to have shorter survival times, attributed to more rapid respiratory decline and accelerated weight loss [30]. Additionally, a large population-based study that categorized patients based on motor and cognitive phenotypes identified notable gender differences. Specifically, it found that older women were more frequently affected by the bulbar phenotype compared to men, suggesting that sex, alongside genetic variants and age, plays a significant role in influencing disease progression [31].

Our analysis did not identify any significant differences in ALS characteristics between urban and rural residents. A large epidemiological study conducted in the United States reported that patients living in rural areas were diagnosed at more advanced stages of the disease and exhibited more severe symptoms at presentation. These findings highlight the potential impact of healthcare accessibility and the need for improved screening and early diagnostic systems in underserved areas [32]. Previous studies suggested that rural environment or living close to agricultural fields may be associated with increased ALS incidence [33,34]. Working in the agricultural sector has also been proposed as a risk factor for ALS [35]. A large meta-analysis observed that the use of pesticides was associated with a faster ALS progression [36]. Another potential aspect that may have an impact on the progression of disease in rural areas would be the presence of old buildings, which are more susceptible for mold growth [37]. Mold and dampness were associated with a higher prevalence of central and peripheric nervous system symptoms in a study conducted on workers from an old Finnish hospital [38].

The median survival time in our cohort was 53 months, which exceeds that reported in a similar Danish cohort study as well as in other studies involving larger populations and longer follow-up periods [39,40,41,42]. One explanation for the increased survival could be the differences in cohort characteristics, such as a younger age at diagnosis, as well as methodological factors, including shorter follow-up duration and small sample size. Studies evaluating cohorts across different time periods have reported a progressive increase in ALS survival, potentially due to earlier diagnosis, the implementation of multidisciplinary care, and improved palliative interventions [43,44,45].

The mean age at diagnosis in our cohort was 56.63 years, with no significant difference between genders, and was lower than the average reported in several other studies [46,47,48,49]. Possible explanations include environmental influences, specific genetic backgrounds, and regional or demographic variations. In our cohort, older age at onset was associated with an increased risk of mortality, although this association did not reach statistical significance, likely due to the relatively small sample size. While numerous studies have validated age at onset as a strong prognostic factor in ALS, indicating that younger patients generally experience longer survival, it remains a somewhat subjective variable [50,51,52].

In our ALS patients, the median diagnostic delay was 9.5 months. Similar diagnostic delays have been reported in other studies [53,54,55]. Interestingly, a longer interval from symptom onset to diagnosis was associated with improved survival in our cohort. This observation is consistent with previous findings that identified diagnostic delay as an independent positive prognostic factor [51,56]. This association may reflect a slower disease progression and milder initial symptoms, which delay patients’ presentation for medical evaluation. Additionally, the clinical heterogeneity of ALS—particularly in cases of limb-onset—can contribute to diagnostic delays, as patients often undergo extensive testing and are initially misdiagnosed [57,58].

Based on the severity, distribution, and progression pattern of signs and symptoms, several ALS phenotypes have been identified, each associated with distinct progression rates and survival outcomes [2]. Bulbar-onset ALS has consistently been linked to shorter survival and more rapid motor decline [59,60]. This poorer prognosis may be partly explained by the more extensive brain tissue loss observed in bulbar-onset cases, as well as the earlier onset of weight loss more frequently reported in this subgroup [61,62]. In our cohort, patients with bulbar-onset exhibited shorter survival times compared to those with spinal-onset, although the difference did not reach statistical significance. Although the trend was clinically meaningful, the small sample size likely limited statistical significance.

Given the variability in clinical presentation, ALS can be classified into distinct subtypes based on the degree and distribution of upper and lower motor neuron (UMN and LMN) involvement. Our analysis revealed that patients with a LMN phenotype at onset—specifically the “flail arm” and “flail leg” variants—had the longest median survival time (71 months), followed by those with the classical ALS phenotype (53 months) and those with bulbar-onset (34 months). Clinical phenotype at onset was significantly associated with survival: patients with bulbar-onset exhibited shorter survival compared to those with LMN-dominant presentations. A previous study observed that patients with LMN-onset demonstrated a more favorable prognosis and were more likely to progress from cervical region to lower thoracic and lumbar segments than to bulbar region [63].

Another study found that the presence of UMN signs at the time of diagnosis in patients with flail arm syndrome has been associated with a poorer prognosis and shorter survival [64]. Colombo et al. assessed the global burden of UMN and found that it independently affected the disease progression and functional disability, but it did not impact survival [65]. In contrast, the degree of LMN involvement appears to be a useful prognostic marker, as prior research has indicated that extensive LMN impairment is associated with a more rapid progression to non-invasive ventilation or death [66]. The authors attributed the diaphragmatic weakness, resulting from the degeneration of cervical lower motor neurons (LMNs), as a key contributing factor to this observation [66].

In addition to the degree of LMN and UMN involvement and their distribution, it is of high importance to analyze the symptom progression model. The spreading direction holds significant prognostic value as the vertical progression pattern was associated with shorter survival periods and the non-contiguous pattern with a more severe upper motor neuron and cognitive involvement [67]. In accordance with previous findings, the disease progression pattern in our research was a strong predictor of prognosis, and patients exhibiting vertical progression had shorter survival times. The bulbar region seems to become involved in late stages in patients with contiguous spread and the non-contiguous pattern is more frequently present in patients with UMN phenotype [68] A caudo-rostral pattern of progression was previously associated with better survival rates and the UMN involvement was associated with accelerated disease progression along a rostral-to-caudal axis, extending from the brainstem to the spinal cord [63,69].

In our cohort, both time to diffusion and time to generalization were identified as significant predictors of survival, as patients with longer intervals had extended survival periods. Although, when integrated into a prognostic model alongside progression pattern, disease progression rate, and clinical phenotype, these parameters did not retain statistical significance. This may be explained by the fact that the included variables bear overlapping prognostic information, particularly as progression patterns may reflect similar features of disease dynamics. Moreover, disease progression rate, which emerged as a stronger predictor, may account for a larger proportion of variance in survival outcomes, diminishing the independent contribution of other variables included in the clinical model. Notably, time to generalization was validated before as a useful independent marker to predict survival in other studies [70,71]. To our knowledge, time to diffusion, defined as the time from the onset of the first symptom to extension to next region, has not been evaluated before as a prognostic marker. Given its ease of clinical assessment, time to diffusion may serve as a valuable prognostic indicator if validated in larger prospective studies.

Among the clinical factors, an extensively studied and validated one has been the disease progression rate, which is a simple and useful marker to predict survival at different time points and stratify patients into distinct prognostic subgroups [19,39,72,73,74]. Consistent with previous studies, disease progression rate was significantly associated with survival in our cohort and emerged as an independent predictor of mortality in the multivariable model. These findings underscore the importance of closely monitoring disease progression and functional status in ALS patients.

Several studies have reported that ALS patients have lower serum 25(OH)D levels compared to healthy controls, and lower 25(OH)D serum levels were associated with poorer cognitive function and increased disease severity [75,76,77,78]. Consistent with previous research, in our study population elevated serum 25(OH)D levels were associated with better survival outcomes. However, in our cohort serum 25(OH)D levels did not demonstrate independent predictive value. Other studies have reported either no significant effect of serum 25(OH)D levels on prognosis or a paradoxical association between higher levels and poorer outcomes [79,80]. A systematic review performed by Lanznaster D et al. identified several methodological limitations in previous studies and found no significant evidence supporting the effect of 25(OH)D on ALS prognosis or any potential therapeutic value [81].

Considering the progression of the disease towards respiratory failure, regular assessment of ventilatory parameters, particularly FVC, has proven to be of great significance in determining the prognosis of ALS patients [1,14,82,83,84,85]. Monitoring the respiratory parameters may help optimize the patient management, especially as non-invasive ventilation has been shown to significantly improve survival outcomes [86]. Consistent with previous findings, our study demonstrated that higher values of FVC, FEV_1_, PEF, and FEF25–75% were significantly associated with more favorable prognostic outcomes, underscoring the relevance of these ventilatory parameters in predicting disease progression. Despite their initial significance, neither FVC nor FEF25–75% demonstrated independent prognostic value when evaluated alongside 25(OH)D and each other, suggesting overlapping contributions to the outcome or insufficient statistical power to detect independent effects.

It was previously suggested that BMI may have clinical significance in the progression of neuroinflammatory and neurodegenerative disorders [87]. Regarding ALS, several studies have explored the influence of nutritional status, with BMI emerging as a significant predictor of patient outcome [87,88]. Moreover, weight loss seems to manifest several years before the onset of motor symptoms [89] and patients with a higher decline in BMI before the first visit to hospital survived for shorter periods, independent of onset age and FVC [90]. In contrast with previous findings, our analysis did not observe a statistically significant association between BMI category and survival. The absence of underweight individuals in our study and the relatively limited sample size within certain subgroups may have reduced the power to detect a meaningful difference.

The nutritional challenges which lead to reduced caloric intake, as well as a hypermetabolic state, represent part of the complex pathophysiological mechanisms through which BMI may influence outcomes of ALS patients [87]. A study which aimed to investigate the optimal caloric intake among individuals with ALS observed that patients whose caloric intake did not exceed 25 kcal/kg (ideal body weight) had a shorter lifespan, and reduced energy intake had an independent negative prognostic value [91].

ALSFRS-R is a validated instrument used to assess the severity of symptoms and functional status [22]. Several studies have shown that the baseline value of this parameter, as well as its rate of decline, holds considerable independent prognostic value and have integrated this scale into different prognostic models [51,52,92,93,94]. While most studies focused on the ALSFRS-R total score or rate of decline, limited attention has been given to the individual subscale items and their potential value in predicting survival [93,95,96]. Considering the multidimensional structure of ALSFRS-R, we analyzed the difference between patient survival based on each subscale. Among these, ALSFRS-R salivation, swallowing, handwriting, and orthopnea were significant prognostic indicators, as patients with higher scores had a significantly reduced hazard of death. Notably, handwriting and swallowing subscales presented independent prognostic power when integrated into a model alongside salivation and orthopnea subscales. Regarding salivation and swallowing, our results may be explained by the fact that dysphagia and sialorrhea are symptoms that suggest bulbar dysfunction, which has previously been associated with poorer survival outcomes [46]. Patients with salivation scores of 3 and 4 had reduced hazards of death compared to those with lower scores (0–2). These findings may be due to the limited number of patients in each subscore category. ALS-FRS-R is an ordinal scale and differences between adjacent subscore categories may not reflect linear changes in clinical risk. Patients with a salivation score of 3 experience a slight excess of saliva. Reporting these symptoms in order to receive conservative interventions may reduce the risk of aspiration pneumonia, a serious complication that can significantly increase mortality. Similarly, orthopnea is an indicator of respiratory impairment, which is a crucial aspect to consider in the disease progression [97]. Orthopnea was previously identified as a prognostic marker, with lower scores linked to a higher chance of requiring non-invasive ventilation [98]. To our knowledge, the role of the ALSFRS-R handwriting subscale as a predictor of survival has not been directly investigated in previous studies. Handwriting is a complex fine motor task that requires preserved muscle strength, as well as intact upper limb coordination and motor planning. Subtle changes in handwriting may precede more evident motor deficits in the disease course and may be more difficult to recognize by patients and clinicians alike in the early stages. Our findings suggest that a decline in the handwriting scale may serve as an early marker of disease progression. It is important to notice that some patients may have constitutionally poor handwriting that has not changed over time, and in those cases a lower score might not indicate ALS progression. Early assessment of handwriting should differentiate patients who maintain previous handwriting function from those who notice a decline, ensuring that any observed deterioration is indeed related to the disease.

Our findings indicate that analyzing individual subscale scores could support a more personalized prognosis. However, the size of our cohort and the design of our study should be considered when interpreting these findings. Although some subscales reached statistical significance, their clinical relevance remains to be confirmed through validation in larger, prospective cohorts.

Strengths of the Study: (1) The follow-up period of three years allowed for robust observation of survival outcomes and assessment of long-term prognostic indicators. (2) The patients underwent a complex clinical and paraclinical evaluation that granted access to multiple and diverse markers to be included in the survival analysis. Limitations of the Study: (1) The study’s sample size, which is relatively small for survival analysis and may limit the statistical power, increasing the risk for Type II errors. As such, the absence of statistical significance for certain variables should be interpreted with caution. The statistical tests and corrections were applied accordingly. (2) Patient recruitment from a single tertiary care center may limit the external validity of our findings to larger ALS populations. Also, the homogeneity of the cohort regarding geographic location and clinical management may restrict the applicability of our findings to more diverse populations. (3) Survival may be influenced by other unmeasured variables, such as dietary habits, physical activity, and medication use.

## 5. Conclusions

In this three-year longitudinal cohort study in ALS patients, we identified several clinical, respiratory, and functional parameters significantly associated with survival. Notably, faster disease progression, vertical progression pattern, and bulbar-onset phenotype were strong predictors of reduced survival. In contrast, better respiratory function at diagnosis, longer time to symptom diffusion and generalization, and preserved bulbar and fine motor function were associated with improved outcomes. Elevated serum vitamin D levels also showed a modest protective effect.

These findings demonstrate the prognostic significance of early clinical, functional, and respiratory evaluations in ALS, and highlight the importance of timely diagnosis and stratification based on disease progression patterns. Such data can inform more personalized patient care, facilitate prognostic discussions, and improve the design of future interventional trials.

## Figures and Tables

**Table 1:** Clinical, paraclinical, and demographic characteristics of the study participants according to gender and environment. F: female gender; M: male gender; ΔPR: the ALSFRS-R progression rate; ALS-FRS: Amyotrophic Lateral Sclerosis Functional Rating Scale; FVC: forced vital capacity; BMI: body mass index; FEV_1_: forced expiratory volume; PEF: peak expiratory flow; FEF2525: forced expiratory flow (25–75). Parametric distribution ^¥^ reported as mean (SD), ° non-parametric distribution reported as median (IQR); ^■^ Welch correction (unequal variances); ^▲^ Fisher’s exact test.

Variable	Total (*n* = 44)	F (*n* = 16)	M (*n* = 28)	*p*	Urban (*n* = 27)	Rural (*n* = 17)	*p*
Age of diagnosis (years) ^¥^	57.97 (12.49)	58.75 (13.65)	57.53 (12.01)	0.76	57.88 (14.03)	58.11 (9.97)	0.95
Age of onset (years) ^¥^	56.63 (12.60)	57.06 (13.81)	56.39 (12.12)	0.86	56.44 (14.32)	56.94 (9.65)	0.89
Onset to diagnosis (months) °	9.5 (5.75–24.25)	14.5 (6–35.25)	9 (5–18.25)	0.26	17 (6–27)	9 (5–15)	0.24
Onset to treatment (months) °	10.5 (5–24.25)	18.5 (6–35.25)	9.5 (5–18.25)	0.14	17 (6–27.5)	10 (5–17)	0.31
Deaths during follow-up	28	11	17	0.74 ^▲^	15	13	0.27 ^▲^
Onset to death (months) °	37 (22–59.25)	44 (20.50–68)	36 (24.25–58.25)	0.93	44 (28–66)	34 (21–57)	0.36
ΔPR °	0.5 (0.33–1.34)	0.4 (0.23–1.47)	0.51 (0.4–0.76)	0.79	0.43 (0.28–1.01)	0.67 (0.42–1.40)	0.31
Beck Depression Inventory °	9 (3.75–14.25)	10 (7–14.25)	8.5 (3–14.50)	0.64	11 (3.14.5)	9 (5–14)	0.87
Frontal Battery Tests °	14 (12–17)	12.5 (16.25–11.5)	15.5 (12–17.25)	0.09	15 (12.5–17)	12 (11–17)	0.19
ALS-FRS °	39 (33–43)	39.5 (31.5–42.25)	39 (33–43.25)	0.64	39 (33–42.5)	41 (33–43)	1.0
BMI ^¥^	25.11 (22.43–27.64)	26.94 (21.53–29.92)	24.96 (23.1–27.23)	0.89	25.24 (22.12–30.08)	24.91 (23.30–26.64)	0.47
Smoking (Yes)	7	7	0	0.73	4	3	1.0
Alcohol consumption (No)	2	2	0	0.52	2	0	0.
Diffusion time (m) ^¥^	11.09 (4.86)	11.93 (4.85)	10.60 (4.89)	0.38 ^■^	12 (8–16)	10 (7–13)	0.23 ^■^
Generalization time (m) ^¥^	19.97 (7.98)	21.37 (7.64)	19.17 (8.20)	0.37 ^■^	23 (15–27)	19 (13–23)	0.20 ^■^
FVC (%) °	101 (49–108.25)	101 (46.5–103.75)	100.5 (65.5–108.25)	0.65	101 (77.5–109.5)	80 (49–101)	0.16
FEV_1_ °	104.5 (52–109)	104.5 (41.75–108)	104 (54.25–109.75)	0.47	106 (72–115.5)	75 (52–105)	0.09
FEV_1_/FVC °	111.5 (105.75–114.25)	112 (104–115)	111 (107.5–114)	0.97	112 (102–114.5)	110 (105–114)	0.42
PEF °	85 (41.5–88)	80.5 (36–86.5)	85 (43.75–88)	0.35	85 (43–88%)	70 (37–88)	0.56
FEF2575 °	102 (49–108)	83 (45.75–102)	102 (51–110)	0.29	102 (51–113)	70 (43–106)	0.22
Vitamin D (ng/mL) °	19.75 (14.8–25.6)	18.10 (14.47–25.8)	20.25 (16.82–25.6)	0.70	21.05 (15.15–26.3)	18.90 (14.30–21.3)	0.61

**Table 2:** Kaplan–Meier survival analysis on the proposed parameters. NA: not available, upper 95CI% could not be calculated due to data censoring or limited number of events; log-rank *p*-values from Kaplan–Meier survival analysis. NA indicates upper CI could not be estimated due to the low number of events.

Variable	Category (*n*)	Median Survival Time (Months), 95% CI	Mean Survival Time (Months), 95% CI	pLog-Rank
**All patients (*n* = 44)**		53 [NA]	33.87 [0.95; NA]	
**Gender**	F (16)	57 [32; NA]	55.65 [41.11; 70.21]	0.9
	M (28)	46.5 [22; NA]	48.04 [34; 16; 61.92]	
**Environment**	U	65 [44; NA]	61.98 [46.96; 77.02]	0.2
	R	34 [22; NA]	44.89 [28.63; 61.17]	
**BMI**	Normal BMI (21)	44 [22; NA]	49.83 [34.14; 65.53]	0.5
	Overweight (15)	60 [34; NA]	52.05 [39.1; 65.0]	
	Obese (8)	NA [36; NA]	72.0 [39.8; 104.2]	
**Age onset**	<50 y.o. (14)	71 [53; NA]	72.64 [50.69; 94.59]	0.07
	≥50 y.o. (30)	36 [25; 71]	44.32 [35; 53.66]	
**ALS subtype**	Bulbar (9)	36 [15; NA]	38.26 [21.07; 55.47]	0.2
	Spinal (35)	57 [32; NA]	59.09 [45.93; 72.27]	
**ALS clinical phenotype**	LMN-predominant (12)	71 [60; NA]	73.42 [51.25; 95.59]	0.02
	Bulbar-onset (7)	34 [14; NA]	28.50 [17.37; 39.63]	
	Classical (25)	53 [24; NA]	47.45 [36.67; 58.23]	
**ALS progression pattern**	Horizontal (23)	71 [60; NA]	74.25 [58.61; 89.91]	0.0002
	Vertical (21)	25 [20; 49]	33.78 [24.02; 43.56]	
**ALSFRS-R**	**Climbing stairs**			0.9
	0 (7)	71 [21; NA]	50.2 [28.8; 71.6]	
	1 (12)	65 [25; NA]	67.9 [44.6; 91.3]	
	2 (7)	44 [32; NA]	52.1 [25.6; 78.5]	
	3 (11)	49 [22; NA]	54.5 [30.9; 78.1]	
	4 (7)	36 [15; NA]	49.1 [12.2–86.1]	
	**Dressing and Hygiene**			0.8
	1 (6)	22 [21; NA]	49.8 [15.8; 83]	
	2 (8)	42 [18; NA]	48.5 [23.3; 73.8]	
	3 (18)	53 [32; NA]	55.8 [40.4; 71.1]	
	4 (12)	49 [36; NA]	58.6 [28.6; 88.6]	
	**Dyspnea**			0.31
	2 + 3 (16)	28.5 [21–NA]	43.43 [29.67–57.21]	
	4 (28)	53 [36–NA]	60.80 [45.2–76.4]	
	**Food and handling utensils**			0.07
	1 (5)	22 [18; NA]	43.4 [11.8; 75.0]	
	2 (11)	22 [20; NA]	36.6 [18.9; 54.2]	
	3 (14)	71 [44; NA]	68.1 [46.9; 89.3]	
	4 (14)	65 [36; NA]	56.4 [42.3; 70.5]	
	**Handwriting**			0.09
	1 + 2 (11)	20 [14; NA]	19.0 [15.8; 22.2]	
	3 (14)	38 [25; NA]	50.0 [30.6; 69.3]	
	4 (19)	71 [49; NA]	65.7 [48.6; 82.7]	
	**Orthopnea**			0.007
	3 (5)	22 [21; NA]	25.8 [13.3; 38.3]	
	4 (39)	60 [36; NA]	56.6 [44.3; 68.9]	
	**Salivation**			0.04
	1 (7)	18 [14; NA]	27.86 [10.75–44.97]	
	3 (10)	71 [32; NA]	66.7 [45.2; 88.2]	
	4 (27)	57 [36; NA]	60.1 [44.3; 75.9]	
	**Speech**			0.2
	0 + 1 + 2 (9)	21 [15; NA]	38.44 [19.68; 57.21]	
	3 (14)	49 [32; NA]	50.6 [33.3; 67.9]	
	4 (21)	65 [36; NA]	67.1 [49.6; 84.6]	
	**Swallowing**			0.04
	2 (7)	15 [11; NA]	30.7 [12.4; 49.1]	
	3 (9)	34 [22; NA]	47.4 [26.3; 68.6]	
	4 (28)	60 [53; NA]	65.4 [50.1; 80.7]	
	**Turning in bed**			0.8
	1 + 2 (12)	22 [22; NA]	49.53 [34.09; 64.97]	
	3 (17)	44 [25; NA]	53.0 [35.2; 70.77]	
	4 (15)	65 [34; NA]	64.0 [40.48; 87.45]	
	**Walking**			0.07
	0 + 1 (7)	71 [36; NA]	61.23 [45.16; 77.31]	
	2 (13)	44 [22; NA]	48.38 [29.23; 67.54]	
	3 (15)	49 [25; NA]	68.36 [45.91; 90.82]	
	4 (9)	36 [22; NA]	46.37 [20.88; 71.86]	

**Table 3:** Summary of the univariate Cox regression test on ALS patients, and Schoenfeld residuals tests. FVC: forced vital capacity; FEV_1_: forced expiratory volume in 1 s; PEF: peak expiratory flow; FEF25–75: mid-expiratory flow; global Schoenfeld residuals test < 0.001.

Variables	Univariate Analysis		Schoenfeld Residuals
	HR. (95% CI)	*p*	*p*
**Gender (M vs. F)**	1.06 [0.49; 2.28]	0.87	
**Environment (Rural vs. Urban)**	1.57 [0.73; 3.38]	0.24	
**Age at onset (years)**	1.033 [0.99; 1.07]	0.06	0.03
**Age at onset stratified** **(<50; ≥50 years)**	2.26 [0.91; 5.59]	0.07	
**BMI**	0.95 [0.87; 1.02]	0.17	
**BMI code**			
**18.5–24.9**	1.55 [0.16; 15.14]	0.70	
**25–29.9**	3.58 [0.45; 28.08]	0.22	
**>29.9**	2.46 [0.30; 19.61]	0.39	
**Onset to diagnosis (months)**	0.97 [0.94; 0.99]	0.02	0.02
**FVC (%)**	0.98 [0.97; 0.99]	0.004	0.08
**FEV_1_**	0.98 [0.97; 0.99]	0.003	0.17
**FEV/FVC**	0.99 [0.95; 1.04]	0.85	
**PEF**	0.98 [0.96; 0.99]	0.01	0.53
**FEF2575**	0.98 [0.97; 0.99]	0.01	0.05
**Vitamin D**	0.92 [0.87; 0.99]	0.02	<0.001
**ΔPR**	3.44 [2.15; 5.51]	<0.001	<0.001
**Beck Depression Inventory**	0.99 [0.94; 1.03]	0.68	
**Frontal Battery Tests**	0.99 [0.92; 1.06]	0.87	
**ALS subtype**	0.58 [0.24; 1.41]	0.23	
**ALS progression pattern**	4.23 [1.87; 9.57]	<0.001	0.005
**ALS clinical phenotype (vs. LMN)**			0.002
**Bulbar-onset**	5.30 [1.53; 18.33]	0.008	
**Classic**	2.18 [0.80; 5.94]	0.12	
**Diffusion Time (m)**	0.84 [0.79; 0.91]	<0.001	0.5
**Generalization time (m)**	0.91 [0.87; 0.95]	<0.001	0.48
**ALS-FRS total**	0.94 [0.88; 1.00]	0.07	
**ALS-FRS stratification**	0.70 [0.33; 1.49]	0.35	
**ALS-FRS-R-Speech**			
**3**	0.82 [0.31; 2.15]	0.69	
**4**	0.47 [0.18; 1.21]	0.12	
**ALS-FRS-R-Salivation**			0.13
**3**	0.30 [0.10; 0.94]	0.03	
**4**	0.36 [0.14; 0.91]	0.03	
**ALS-FRS-R-Swallowing**			0.01
**3**	0.60 [0.20; 1.78]	0.36	
**4**	0.32 [0.12; 0.82]	0.01	
**ALS-FRS-R-Handwriting**			0.04
**3**	0.80 [0.32; 1.98]	0.64	
**4**	0.37 [0.14; 0.98]	0.04	
**ALS-FRS-R-Food and handling utensils**			
**2**	1.22 [0.38; 3.91]	0.73	
**3**	0.41 [0.11; 1.42]	0.16	
**4**	0.46 [0.13; 1.60]	0.22	
**ALS-FRS-R-Dressing and hygiene**			
**2**	0.93 [0.26; 3.32]	0.91	
**3**	0.69 [0.22; 2.15]	0.52	
**4**	0.61 [0.16–2.32]	0.47	
**ALS-FRS-R-Turning in bed**			
**3**	1.06 [0.44; 2.54]	0.89	
**4**	0.74 [0.27; 2.03]	0.57	
**ALS-FRS-R-Walking**			
**2**	1.78 [0.59; 5.33]	0.30	
**3**	0.98 [0.31; 3.13]	0.98	
**4**	1.79 [0.53; 6.05]	0.34	
**ALS-FRS-R-Climbing stairs**			
**1**	0.66 [0.20; 2.08]	0.48	
**2**	1.24 [0.36; 4.29]	0.73	
**3**	0.94 [0.31; 2.83]	0.91	
**4**	1.12 [0.30; 4.09]	0.86	
**ALS-FRS-R-Orthopnea**	0.22 [0.09; 0.73]	0.01	0.44
**ALS-FRS-R-Respiratory Insufficiency**	0.23 [0.05; 1.10]	0.07	
**ALS-FRS-R-Dyspnea**	0.67 [0.31; 1.43]	0.30	
			<0.001

**Table 4:** Multivariate COX regression model with Firth’s correction for survival analysis in ALS patients. FVC: forced vital capacity; FEF25–75: mid-expiratory flow; LMN: lower motor neuron; UMN: upper motor neuron.

Model	Predictors	Coefficient	HR	95CI%	*p*	Wald Test (*p*)	Likelihood Ratio-Test (*p*)
Model 1	Age at onset	0.03	1.03	[0.99; 1.07]	0.05	0.02	0.01
	Onset to diagnosis (m)	−0.02	0.97	[0.94; 0.99]	0.02		
Model 2	FVC	0.01	0.99	[0.96; 1.01]	0.47	0.04	0.03
	FEF 25–75	0.01	0.99	[0.97; 1.02]	0.80		
	Vitamin D	0.03	0.96	[1.04; 0.7]	0.40		
Model 3	Progression pattern					<0.001	<0.001
	*Vertical*	0.99	2.69	[0.90; 9.16]	0.07		
	Clinical phenotype						
	*Bulbar*	−0.49	0.60	[0.09; 3.61]	0.58		
	*Classic*	−0.43	0.64	[0.14; 2.57]	0.53		
	ΔPR	1.05	2.86	[1.86; 4.68]	<0.001		
	Diffusion time	−0.20	0.81	[0.62; 1.06]	0.12		
	Generalization time	0.02	1.02	[0.86; 1.20]	0.75		
Model 4	ALS-FRS-R Salivation					0.01	0.002
	*3*	−0.87	0.41	[0.10; 1.66]	0.20		
	*4*	0.17	1.18	[0.28; 4.49]	NA		
	ALS-FRS-R Swallowing						
	*3*	−1.23	0.29	[0.07; 1.18]	NA		
	*4*	−1.83	0.16	[0.04; 0.71]	0.01		
	ALS-FRS-R Orthopnea						
	*4*	−1.09	0.36	[0.11; 1.22]	0.09		
	ALS-FRS-R Handwriting						
	*3*	−0.31	0.73	[0.22; 2.53]	0.61		
	*4*	−1.66	0.19	[0.04; 0.78]	0.02		

## Data Availability

No new data were created or analyzed in this study.

## Abbreviations

The following abbreviations are used in this manuscript:

ALS	Amyotrophic Lateral Sclerosis
ALSFRS-R	Revised ALS functional rating scale
FVC	Forced vital capacity
FEV_1_	Forced expiratory volume in one second
PEF	Peak expiratory flow
FEF25–75	Forced expiratory flow 25–75
ΔPR	Disease progression rate
BMI	Body mass
LMN	Lower motor neuron
UMN	Upper motor neuron
HSP	Horizontal spreading pattern
VSP	Vertical spreading pattern
FAB	Frontal Assessment Battery
BDI	Beck Depression Inventory
25(OH)D	25-hydroxivitamin D
